# Diagnostic comparison of malaria infection in peripheral blood, placental blood and placental biopsies in Cameroonian parturient women

**DOI:** 10.1186/1475-2875-8-126

**Published:** 2009-06-08

**Authors:** Judith K Anchang-Kimbi, Eric A Achidi, Blaise Nkegoum, Eva Sverremark-Ekström, Marita Troye-Blomberg

**Affiliations:** 1 Department of Plant and Animal Sciences, University of Buea, PO Box 63 Buea, Cameroon; 2 Faculty of Health Sciences, University of Buea, Cameroon; 3 Department of Anatomy and Pathology, University of Yaoundé Teaching Hospital, Cameroon; 4 Department of Immunology, Wenner-Gren Institute, Stockholm University, Sweden

## Abstract

**Background:**

In sub-Saharan Africa, *Plasmodium falciparum *malaria in pregnancy presents an enormous diagnostic challenge. The epidemiological and clinical relevance of the different types of malaria diagnosis as well as risk factors associated with malaria infection at delivery were investigated.

**Method:**

In a cross-sectional survey, 306 women reporting for delivery in the Mutenegene maternity clinic, Fako division, South West province, Cameroon were screened for *P. falciparum *in peripheral blood, placental blood and placental tissue sections by microscopy. Information relating to the use of intermittent preventive treatment in pregnancy with sulphadoxine/pyrimethamine, history of fever attack, infant birth weights and maternal anaemia were recorded.

**Results:**

Among these women, *P. falciparum *infection was detected in 5.6%, 25.5% and 60.5% of the cases in peripheral blood, placental blood and placental histological sections respectively. Placental histology was more sensitive (97.4%) than placental blood film (41.5%) and peripheral blood (8.0%) microscopy. In multivariate analysis, age (≤ 20 years old) (OR = 4.61, 95% CI = 1.47 – 14.70), history of fever attack (OR = 2.98, 95% CI = 1.58 – 5.73) were significant risk factors associated with microscopically detected parasitaemia. The use of ≥ 2 SP doses (OR = 0.18, 95% CI = 0.06 – 0.52) was associated with a significant reduction in the prevalence of microscopic parasitaemia at delivery. Age (>20 years) (OR = 0.34, 95% CI = 0.15 – 0.75) was the only significant risk factor associated with parasitaemia diagnosed by histology only in univariate analysis. Microscopic parasitaemia (OR = 2.74, 95% CI = 1.33–5.62) was a significant risk factor for maternal anaemia at delivery, but neither infection detected by histology only, nor past infection were associated with increased risk of anaemia.

**Conclusion:**

Placenta histological examination was the most sensitive indicator of malaria infection at delivery. Microscopically detected parasitaemia was associated with increased risk of maternal anaemia at delivery, but not low-grade parasitaemia detected by placental histology only.

## Background

Malaria due to *Plasmodium falciparum *infection during pregnancy is a serious public health problem in sub-Saharan Africa. One woman in four has evidence of placental infection at the time of delivery [1,2]. It is well known that infection with malaria during pregnancy leads to the selective adherence of infected erythrocytes (IEs) in the placenta [3-5]. *Plasmodium falciparum *erythrocyte membrane protein 1 (*var2csa *PfEMP1) is the principal chondroitin sulfate A (CSA) binding ligand mediating placental sequestration of IEs [6]. Consequently higher numbers of IEs containing mature trophozoite and schizont stage parasites may be found in the placenta [4], to higher densities than in the peripheral circulation. Sequestration of parasites in the intervillous spaces (IVS), contributes to maternal morbidity, low birth weight, and preterm delivery [1,7]. Moreover, malaria in pregnancy is an important cause of severe anaemia in pregnant African women [8].

Placental malaria (PM) is one of the major features of malaria during pregnancy and has been widely used as a standard indicator to characterize malaria infection in epidemiologic investigations [9]. A number of factors influence the prevalence of malaria in pregnant women, including maternal age, gravidity, use of prophylaxis, nutrition, host genetics, level of anti-parasite immunity, as well as parasite genetics and transmission rates [10]. The prevalence and risk factors of placental malaria in sub-Saharan Africa have been assessed based on different definitions of placental malaria using diverse techniques of sample collection and analysis [9]. Some studies based their definition on the presence of malaria parasite and/or pigments in blood smears from placental blood [10-13]. Others based their definition on histological findings [7,14-16], histidine-rich-protein-2 (HRP2) capture test [17,18] and polymerase chain reaction (PCR) [18-20].

Placental histology is considered the "gold standard" of malaria diagnosis in pregnancy for epidemiological or biological study purposes, because it can show signs of active (presence of IEs only in the IVS), active chronic (presence of IEs and pigment in monocytes) or past (malarial pigment in fibrin) infections [21,22]. However, due to limited technical expertise, such testing is rarely available in endemic areas. Most studies in sub-Saharan Africa have relied on the results of the placental blood smear [9], the sensitivity of which is low compared with placental histopathology [16].

This study: i) determined and compared malaria infection at delivery in maternal peripheral blood, placental blood and placental histological sections; ii) assessed the epidemiological and clinical significance of *P. falciparum *infections detected by blood smear microscopy, histology only and past infection, as well as risk factors associated with malaria infection at delivery.

## Methods

### Study area and population

The study was conducted in the Mutengene health area from March to October 2007. Mutengene is a semi-urban, road-junction town, located in the Mt Cameroon region, Fako Division of the South West Province of Cameroon. The Mutengene medical centre is the only government-owned institution that offers antenatal care, preventive and curative services at affordable cost in the health area. This town is located at about 220 m above sea level and has a heterogeneous population of approximately 40,000 inhabitants who originate mainly from neighbouring provinces in search of its fertile farmland and business opportunities [11,23].

The Mt Cameroon region has an equatorial climate made up of a long rainy season that starts in March and ends in October with maximum rainfall in August and September. The dry season starts in late October and ends in February. The mean value of the minimum temperature varies between 20.0°C in December and 18.0°C in August and the mean value of the maximum temperature ranges between 35.0°C in February and 30.0°C in March [23]. Mutengene is characterized by mean temperatures of 25.1°C and mean relative humidity of 83.1% (Cameroon Development Corporation (CDC) weather records, 2004).

The Mt Cameroon region is hyperendemic for malaria, with *P. falciparum *being the predominant malaria parasite species [24]. A previous study on malaria in pregnancy in Mutengene from 1998–2001 recorded malaria parasite rates of 32.7%, 33.7% and 7.8% in maternal, placental and cord blood respectively [13]. In addition, this study showed that anaemia is a major health problem in this area and malaria contributes about 50% of the anaemic cases [11]. Intermittent preventive treatment in pregnancy (IPTp), using regular treatment doses of the antimalarial sulphadoxine/pyrimethamine (SP) was introduced as a national policy in Cameroon in 2003 and implementation is still in progress [25]. The study received ethical clearance from the Delegation of Public health, Buea, South West Province.

### Sample collection and processing

Pregnant women reporting for delivery at the Mutengene maternity health centre and who consented to participate in the study were enrolled. Expectant mothers with evidence of chronic illness and complicated pregnancy (hypertension, preeclampsia, diabetes) were not eligible and cases of multiple pregnancies were excluded. All maternal and infant characteristics (age, gravidity, gestational age, birth weights, number of antenatal visits, SP dosage, and history of fever attack during pregnancy) were documented using a standard questionnaire. IPT/SP during pregnancy is administered during antenatal visits under the supervision of a midwife who records and signs in patients' antenatal case report book. SP dosage and compliance and history of fever attack were confirmed from the patient's medical record book and by personal interview. Human immunodeficiency virus (HIV) infection status was known for mothers recruited. Women in this setting are offered routine confidential HIV testing at first antenatal clinic (ANC) visit, but testing was done at delivery for women who had no antenatal follow-up or had enrolled in maternity clinics out of the study area with no ANC cards. We did not obtain informed consent to publish data on HIV status of the participants.

Maternal peripheral venous blood (2 ml) was collected by venopuncture within 24 hours of parturition and used to prepare thick blood films. Immediately following delivery, the placenta was obtained and a small piece of placental tissue (0.5 cm^3^) excised from the centre of the placenta and used to prepare impression smears. A larger biopsy specimen of placental tissue (2 by 2 by 1 cm) was fixed in 10% neutral buffered formalin for histopathological studies.

### Parasitological examination

Placental impression smears were fixed in methanol and together with thick films stained for 20 mins with 5% Giemsa (Sigma). Fixed placental biopsies were transferred to the Anatomy and Pathology (ANAPATH) Laboratory, University of Yaoundé I Teaching Hospital (CHU), Yaoundé, Cameroon, where they were processed, embedded in paraffin wax and sectioned onto slides by standard techniques. Sections were later stained with haematoxylin-eosin stain for detection of active and past infections. Microscopic examination of blood smears was done under oil immersion for parasite detection and 200 high power fields were examined before the smear was considered negative. Parasites were counted against 200 leucocytes assuming an average leucocyte count of 8,000 per microlitre of blood [26]. To determine the percentage of malaria parasitaemia from placental impression smears, malaria parasite-infected red cells were counted against 1,000 erythrocytes.

Placental histological sections were first examined by the one of the investigators (JKA) and later confirmed by a more experienced histopathologist (BN) without knowledge of the blood film microscopy results. One thousand intervillous cells (IVS) were counted to determine the level of parasitaemia in placenta tissue sections. Past infection was defined as the presence of malaria pigment in fibrin or monocyte/macrophage without malaria parasites [14]. Histology was re-examined in all cases in which histology and blood film results were in disagreement. Sections were observed under polarised light to assess the presence of malaria pigment [27].

### Haematological analysis

Heparinized, microhaematocrit tubes were filled with a sample of peripheral blood and centrifuged using a microhaematocrit centrifuge. The haematocrit (packed cell volume (PCV) was read using haematocrit reader (Hawksley). Haemoglobin concentration was calculated from PCV values as described by Topley [28]. Anaemia was defined as Hb < 11.0 g/dl [29].

### Statistical analyses

All data were entered and analysed using SPSS version 11.5. Malaria infection at delivery was classified as microscopic parasitaemia detected by blood smear microscopy, parasitaemia by histology only, past infection and no malaria. Associations of the different types of malaria diagnosis with age group, parity, use and doses of IPTp-SP and history of fever attack were evaluated using Pearson Chi-Square (χ^2^) test, odds ratio (OR) test in univariate analyses and logistic regression in multivariate analyses. Differences in group means were assessed using ANOVA, Mann-Whiney U test or Kruskall Wallis test. Age and parity was categorized as follows: age (≤ 20, 21–25, >25) years and parity (primiparae, secundiparae and multiparae (≥ 3 pregnancies), use of SP (no SP, 1 dose and ≥ 2 doses), fever history (fever attack = Yes, No fever attack = No). Statistical significance was set at p ≤ 0.05.

## Results

### Description of study participants

A total of 306 women delivering at the Mutengene matenity ward were recruited. The mean age of the mothers was 23.8 ± 5.2 (range; 14–40) years, where primiparae (19.5 ± 2.4) were significantly younger (p < 0.001) compared with secundiparae (22.6 ± 3.0) and multiparae (28.2 ± 4.6). Among the women, 90.1% (272/302) reported taking IPTp-SP during pregnancy. For women with number of doses recorded, 47.8% (129/270) had taken one dose, 52.2% (141/270) took two or more doses and 9.9% (30/302) did not take SP during the entire pregnancy period. Peripheral blood was collected from 287. Reported history of fever was 43.6% (125/287). One hundred and seventy-six (63.3%; 278) women were anaemic at delivery and the prevalence of low birth weight was 5.6% (17/306). The prevalence of HIV infection among women recruited was 5.6%; (17/306).

#### Detection and prevalence of malaria infection

Sixteen women (5.6%; 16/287) had malaria parasite infection diagnosed by peripheral blood microscopy. Seventy-eight (25.5%; 78/306) had placental infection diagnosed by placental blood smear examination. Of the 25% diagnosed by placental blood smear, 19.6% (60/306) were with active infection while 5.9% (18/306) had past infection. Placental histological examination detected 185 (60.5%, 185/306) placental malaria positive women. Of these, 33.7% (103) had active placental infections while 26.8% (82) had past infection. Overall, 39.2% (120/306) had active *P. falciparum *infection, defined as the presence of malaria parasites as detected in thick peripheral blood, placental impression blood films or placental tissue sections. Of the active infections, 44.2% (53) were detected by histology only and 55.8% (67) by blood smear microscopy and histology. Of the 82(26.8%) women with past infection on histology, 12 were positive for malaria parasites on blood smear microscopy (3 positive on peripheral blood and 9 on placental blood) thus the overall prevalence of past infection was 22.9% (70). The cumulative prevalence of malaria infection at delivery (total number of women positive for malaria either by histology or peripheral and placental blood smear examination was 62.1% (190/306).

**Figure 1:**
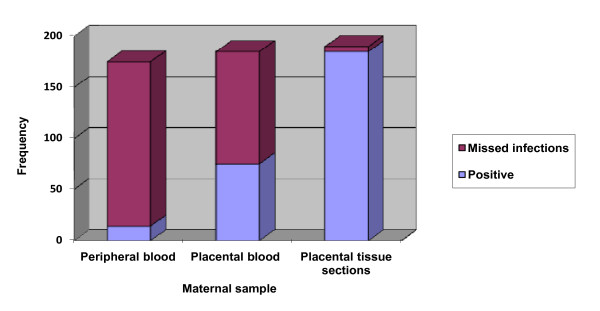
**Frequency distribution of malaria infection in peripheral blood, placental blood and placental tissue sections**. Malaria infection was defined as presence of malaria parasites, malaria pigment (haemozoin) in monocytes and/or fibrin detected by microscopy in maternal peripheral blood, placental blood and placental tissue sections from parturient women.

Placental histology detected all but three of the placental infections detected by placental blood smear examination, as well as all but two of the malaria infection detected by peripheral blood smear; giving a sensitivity of 97.4%. One hundred and ten women with histological evidence of placental infection were missed on placental blood smear, which had a sensitivity of 41.5%. One hundred and sixty-one women positive by placental histology were missed on peripheral blood examination, which had a sensitivity of 8% (Figure 1). The overall prevalence of placental malaria at delivery was 61.4% (188/306). There was a significant positive correlation between parasitaemia levels determined by placental blood smear and tissue sections (r = 0.757; p < 0.001). Among the women in whom malaria parasite was detected by histology only, the median (range) placental parasite density was 0.38% (0.10 to 3.2%) lower (p < 0.001) compared to that for women with positive placental blood smears (0.83%; range, 0.11 to 57.27%). Generally, placental parasites were usually scarce: 66.1% (76/115) of actively infected placentas had parasitaemia less than 1% whereas only 12.2% (14/115) showed 10% or more parasitized erythrocytes.

### Factors influencing the prevalence of malaria infection at delivery

Microscopically detected parasitaemia was the only clinically relevant malaria infection at delivery and factors influencing its presence were analysed (Table 1). In multivariate analysis, younger age (≤ 20 years) and reported fever history were independent risk factors associated with microscopic parasitaemia while the use of ≥ 1 SP doses was associated with reduction in the prevalence of infection. Parity was not associated with microscopic infections neither in univariate nor multivariate analyses. Nevertheless, past infections were common (χ^2 ^= 6.328; p = 0.042) in primiparous (31.6%) compared to secundiparous (21.1%) and multiparous (17.4%) women. The presence of infections detected by histology only (low-grade parasitaemia) was significantly associated with age (>20 years) (OR = 0.34, 95% CI = 0.15 – 0.75) where infections detected by histology only were more common (χ^2 ^= 7.616; p = 0.006) in women > 20 years old (20.8%) than in those ≤ 20 years (8.5%). Parity, SP dosage, or fever history were not associated with the prevalence of low-grade infection.

**Table 1:** Risk factors of microscopic parasitaemia

**Factors**	**% positive**	**Univariate analysis**		**Multivariate analysis**	
		OR (95% CI)*	*P*	OR (95% CI) **†**	*P*
**Age (years)(n)**					
**≤ 20 (94)**	31.9 (30)	2.69 (1.34 – 5.41)	0.005	4.61 (1.47 – 14.70)	0.01
**21–25 (108)**	18.5 (20)	1.30 (0.63 – 2.71)	0.478	2.03 (0.80 – 5.20)	0.137
**>25 (101)**	14.9 (15)	Reference			
					
**Parity (n)**					
**I (95)**	29.5 (28)	1.78 (0.95 – 3.35)	0.072	0.61 (0.22 – 1.69)	0.337
**II (90)**	17.8 (16)	0.92 (0.46 – 1.87)	0.820	0.40 (0.15 – 1.06)	0.064
**≥ III (121)**	19.0 (23)	Reference			
					
**SP dosage (n)**					
**≥ 2 (141)**	14.9 (21)	0.26 (0.11 – 0.62)	0.002	0.18 (0.06 – 0.52)	0.002
**1 (129)**	24.0 (31)	0.47 (0.21 – 1.09)	0.076	0.31 (0.11 – 0.88)	0.028
**No SP (30)**	40.0 (12)	Reference			
					
**Fever history (n)**					
**Yes (125)**	28.8 (36)	2.45 (1.36 – 4.40)	0.002	2.98 (1.58 – 5.73)	0.001
**No (162)**	14.2 (23)	Reference			

OR = odds ratio; CI = confidence interval

* Estimated by Pearson-Chi-Square

† Estimated by multivariate logistic regression including all risk factors in the table

### The effect of malaria infection status at delivery on infant birth weight and maternal anaemia

The mean birth weights of babies did not differ with malaria infection status, while mean haemoglobin concentrations were significantly lower in women with microscopically detected infection compared to uninfected women (Table 2). Similarly, microscopic parasitaemia (OR = 2.74, 95% CI = 1.33–5.62) was a significant (p = 0.006; 49/62) risk factor for maternal anaemia at delivery, but low-grade parasitaemia (OR = 0.61, 95% CI = 0.31–1.22; p = 0.162; 22/48) and past infections (OR = 1.73, 95% CI = 0.89 – 3.39; p = 0.106; 43/61) did not significantly increase the risk of anaemia.

**Table 2:** Comparison of birth weight (kg) and maternal haemoglobin levels (g/dl) according to presence or absence of malaria infection as detected by microscopy and histology

Infection classification^*a*^	Birth weight (kg) (± SD)	Hb (g/dl) (± SD)
Malaria on microscopy	3.18 ± 0.54 (67)^*b*^	9.59 ± 1.87 (62)***^c^
Malaria on histology only	3.40 ± 0.49 (53)^*d*^	10.90 ± 1.38 (48)^*e*^
Past malaria	3.22 ± 0.52 (70)^*f*^	10.33 ± 1.29 (61)^*g*^
No malaria	3.31 ± 0.12 (116)	10.57 ± 1.67 (107)

*** Significant

^*a *^Malaria on microscopy: parasites identified by peripheral and placental blood

microscopy; malaria on histology only, parasites identified by histology only but not blood smear microscopy; past malaria infection, malaria pigment identified by histology with negative blood film microscopy; no malaria, no parasites by blood smear microscopy nor placental histology.

^*b *^P = 0.09 ^*c *^P < 0.001, ^*d *^P = 0.283, ^e ^P = 0.229, ^*f *^P = 0.214, ^*g*^*p = 0.346 * represent comparison with placentas with no *P. falciparum *infection.

## Discussion

This study determined the prevalence of malaria infection and parasite density in Cameroonian parturient women using maternal peripheral blood microscopy, placental blood microscopy and placental histology. The epidemiological and clinical relevance of the different types of malaria diagnosis as well as some risk factors associated with malaria infection were investigated.

Overall, pregnant women in this semi-urban area of Cameroon were frequently (62.1%) exposed to *P. falciparum *infection during pregnancy. Using blood smear microscopy, 21.9% of the women were found to harbour malaria parasites at delivery similar to findings reported in Yaoundé [17,19]. Malaria parasitaemia rate increased to 39.2% when the results of placental histology were added. Furthermore, microscopy of peripheral blood missed more microscopic placental infections, with 49 out of 60 women having a negative peripheral blood film. The observed sensitivity of placental histology is in accord with previous reports [15,16,30] and emphasis the gross underestimation of placental malaria infection by peripheral blood microscopy in pregnant women living in endemic areas. It is possible that peripheral parasitaemia may remain below the levels of microscopic detection while parasites may be harboured by the placenta and evade circulation. Nevertheless some studies have reported high sensitivities of peripheral blood immunochromatographic (ICT) strip test that detects *P. falciparum *histidine-rich protein 2 (HRP-2) [17,31] and PCR, that amplifies *P. falciparum*-specific DNA for detection of low-level parasitemia or circulating genetic material [32] compared to peripheral blood microscopy in detecting microscopically confirmed placental *Plasmodium falciparum *[19,31]. Malaria in pregnancy has significant adverse effects on the mother and foetus. Consequently, early and accurate diagnosis of malaria in pregnancy is absolutely imperative. Obtaining results quickly from the examination of blood samples from pregnant women with suspected malaria is now made possible by the use of rapid malaria diagnostic tests (RDTs), although their use in developing countries is limited by their high cost and availability [33].

There was a significant positive correlation between parasitaemia levels determined by placental blood smear and tissue sections (r = 0.757; p < 0.001), the median placental parasitaemia levels being higher in placental blood smears (0.83%) compared to that detected in placental tissue sections (0.38%). Two concepts are suggested. Firstly, impression smears correlate quite well with histology. Secondly, infection detected only by histology is lower level and potentially less significant than infection found on impression smears.

It is well established that primigravidae are indisputably at greater risk of malaria infection during pregnancy in stable malaria regions [34]. In the present study, parity was associated only with past infections. Similar to previous studies, age (≤ 20 years old) was an independent risk factor for microscopic parasitaemia after adjusting for parity [10,35-38]. Furthermore, parity had no effect on the prevalence of low grade parasitaemia in pregnant women similar to findings reported elsewhere [16,19] but women > 20 years had higher prevalence of low-grade parasitaemia than women ≤ 20 years old). This suggests that age-associated immunity may play an important role in limiting *P. falciparum *to low parasite densities in areas of high and stable transmission [39]. The relationship between the role of age as a determinant of immunity and vaccine studies is not really clear. It is suggested that non-pregnancy-specific vaccines might help teenage mothers [40], but at present it is not clear whether vaccines like RTS, S is meant rather than VAR2CSA vaccines.

It is generally assumed that in areas of stable transmission, parasitaemic pregnant women are rarely symptomatic, and that severe disease or death from malaria is extremely unusual [41]. However, this assumption has been based on few studies and symptoms suggestive of malaria among pregnant women attending maternity clinics are frequent [42]. Headache, arthromyalgias and history of fever are the most common symptoms reported in pregnant women [42]. In this study, history of fever attack was common (44%) in women during pregnancy and was associated with approximately three-fold increased risk of microscopic parasitaemia at delivery. Future studies are needed to evaluate the clinical presentation of malaria episodes during pregnancy in stable transmission areas to better control the disease. Secondly, the lack of appropriate or timely treatment of symptomatic malaria in pregnancy may lead to adverse pregnancy outcome.

Women with blood smear microscopically detectable malaria were likely to be anaemic and had a mean haemoglobin concentration of 0.98 g/dl lower than that of uninfected women. Similar to findings reported elsewhere [16,19,31] blood film microscopy identified women at highest risk of anaemia. This study did not show an association between infection detected by histology only and anaemia nor a change in mean haematocrit levels. Equally, several studies, [16,19,37] failed to obtain an association between subpatent *P. falciparum *infection in both peripheral and placental blood and increased risk of anaemia. On the contrary, in Ghana, as reported by Mockenhaupt *et al *[31,43], submicroscopic infections were found to be a risk factor for maternal anaemia. The impact of subpatent malaria infections on anaemia might vary with different malaria endemicity levels and therefore with the level of pre-pregnancy acquired malaria immunity [37]. Anaemia may be caused by an increase in density of parasitaemia rather than the mere presence of parasite infection.

In Malawi, Rogerson *et al *[16] showed that birth weights of newborns differed significantly with a diagnosis of malaria. Further, the prevalence of LBW babies was significantly higher for women with blood film microscopically detectable infection compared to those with no parasites by blood film microscopy or placental histology. The prevalence of LBW in the study area was particularly low thus it is possible that the small sample size did not provide the power needed to detect the effect of malaria on LBW. In other areas, LBW among women with malaria infection is frequently around 20% and close to 10% in those without [16]. This may explain the lack of associations. Placental monocytes and fibrin containing malaria pigment have been shown to play important roles in the pathogenesis of pregnancy-associated malaria [22]. There is the need for prospective studies in the study area that will examine the causal roles of monocyte and fibrin containing pigment in the pathogenesis of decreased birth weight and maternal anaemia.

It is not surprising that women who reported to have received one or more doses of SP had a decreased risk of microscopic parasitaemia at delivery than those who had no SP during pregnancy. Furthermore, two or more doses of SP significantly reduce the prevalence of microscopic parasitaemia among pregnant women similar to findings of previous studies [44,45]. The prevalence of peripheral malaria parasitaemia (5.6%) and placental malaria parasitaemia (25.5%) as detected by blood smear microscopy is low compared to that previously reported in the same community where peripheral and placental malaria parasitaemia (33.2% and 33.6% respectively) were similar [13]. This study was carried out from 1998–2001 when pyrimethamine chemoprophylaxis had been recommended for use in pregnancy. It can be observed from the current study that maternal peripheral and placental malaria infection at delivery has decreased in this area confirming that IPT with SP may be effective in reducing malaria parasitaemia in pregnancy after implementation. Similar to findings reported elsewhere [35,45-47]. Approximately half of the women in the study received ≤ 1 SP dose. Thus a study to evaluate the coverage of IPTp-SP in this area in order to identify facilitating factors and operational challenges for scaling up IPT delivery is imperative.

## Conclusion

In summary, malaria in pregnancy was most sensitively diagnosed by placental histology. Younger age and history of fever attack during pregnancy were independent significant risk factors associated with microscopic parasitaemia at delivery. Blood smear microscopy identified a subset of women with malaria who had a high risk of anaemia whereas low-grade parasitaemia detected by histology only and past infection were not associated with maternal anaemia at delivery. Improving IPTp-SP coverage and adherence will substantially reduce malaria parasitaemia and maternal anaemia in pregnant women in this area. Proper diagnosis as well as ensuring appropriate and timely treatment of symptomatic malaria in pregnancy is recommended.

## Competing interests

The authors declare that they have no competing interests.

## Authors' contributions

JKAK: Conception and design of study, data collection, analysis, interpretation and manuscript preparation. EAA: Supervision and critically reading of manuscript for important and intellectual content. NB: Contributed in placental pathohistological studies. ESE: Design of study and critically reading of manuscript for important and intellectual content; MTB: Supervision and revision of manuscript. All authors read and approved the manuscript.
